# Supplementary figures and images for: Multifunctional molecular hybrid for targeted colorectal cancer cells: Integrating doxorubicin, AS1411 aptamer, and T9/U4 ASO
Source: PLoS One. 2025 Feb 13;20(2):e0317559. doi: 10.1371/journal.pone.0317559 (PMC11825018; doi:10.1371/journal.pone.0317559)

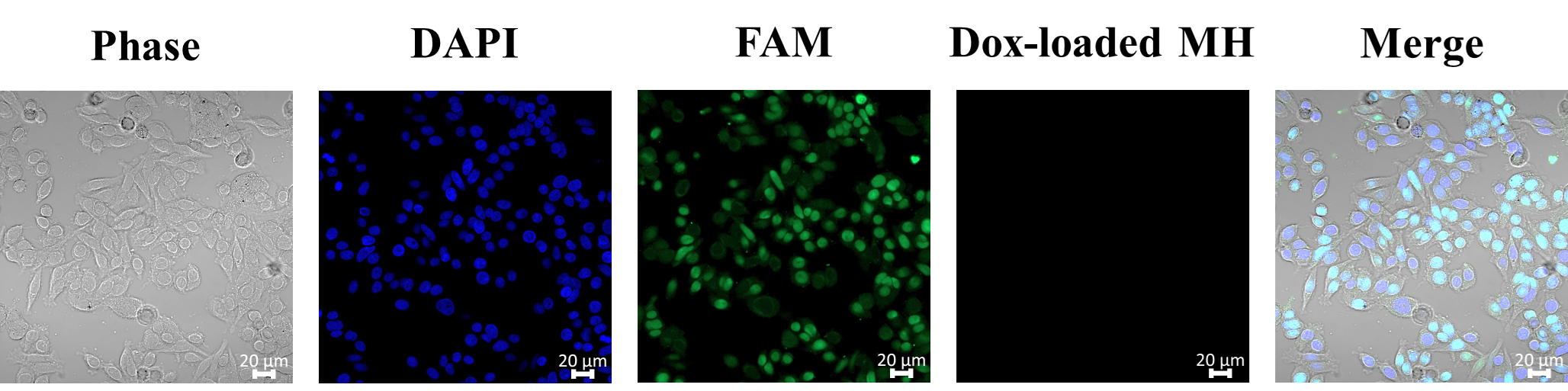

Supplement: S1 Fig — To assess the intercalation of Dox into dsDNA SW480 cells were seeded in 8-well chamber slide at density 9x10^5^ and incubated 24 h. After, the cells were treated with 10 μM Dox-loaded AS-T9/U4_MH for 1.5 h. After treatment, the cells were washed twice with PBS and stained with DAPI to visualize the nuclei. Subsequently, the cells were imaged using a CLSM. (TIF) [file pone.0317559.s001.tif]

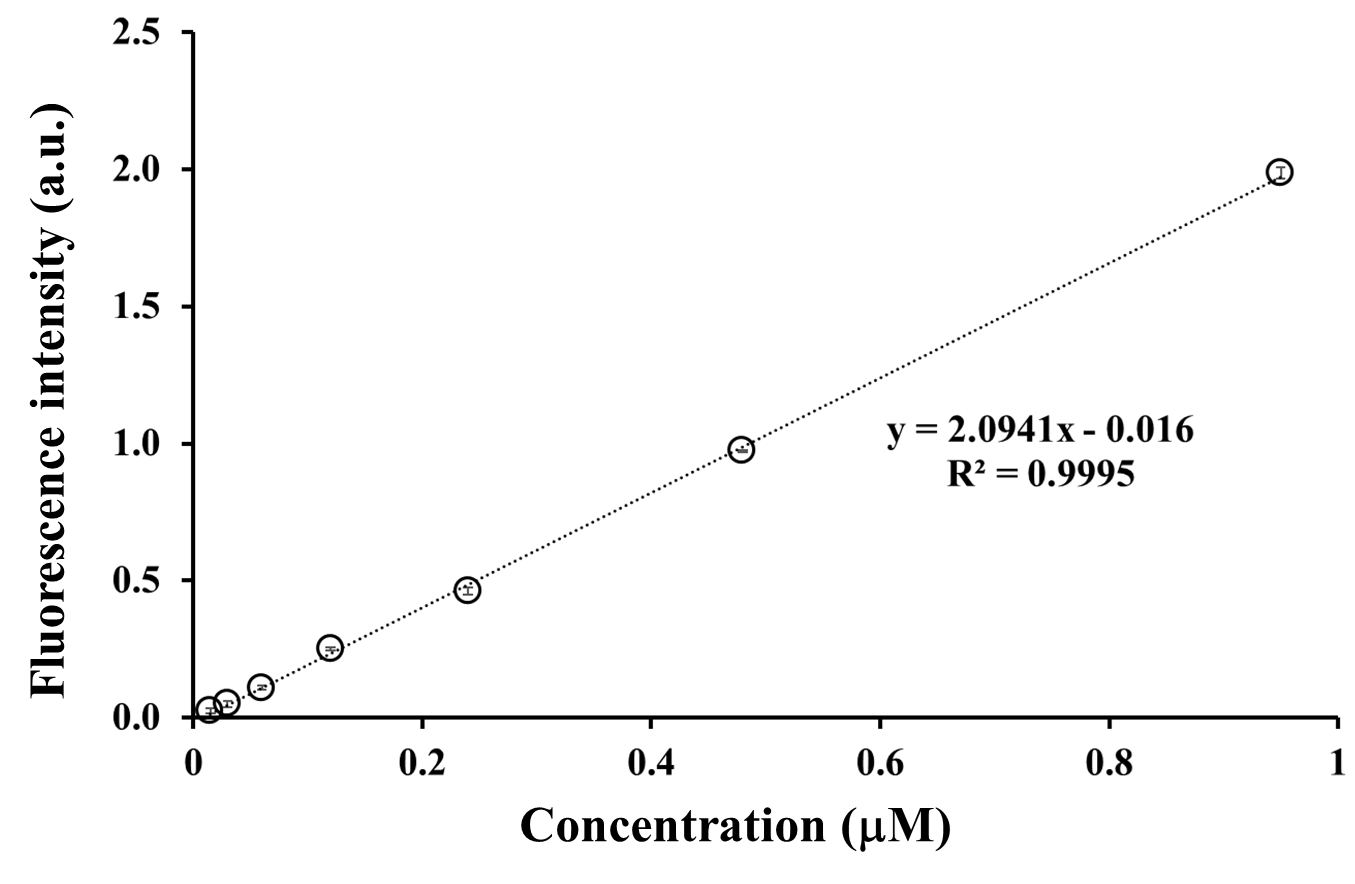

Supplement: S2 Fig — Standard curve was created to evaluate the concentration of Dox remaining in the solution after intercalation. Dox solutions at various concentrations, including 0.95, 0.48, 0.24, 0.12, 0.06, 0.03, and 0.015 μM, were prepared. The fluorescence intensity at 590 nm was measured when excited at 480 nm using a Virokcan microplate reader. (TIF) [file pone.0317559.s002.tif]

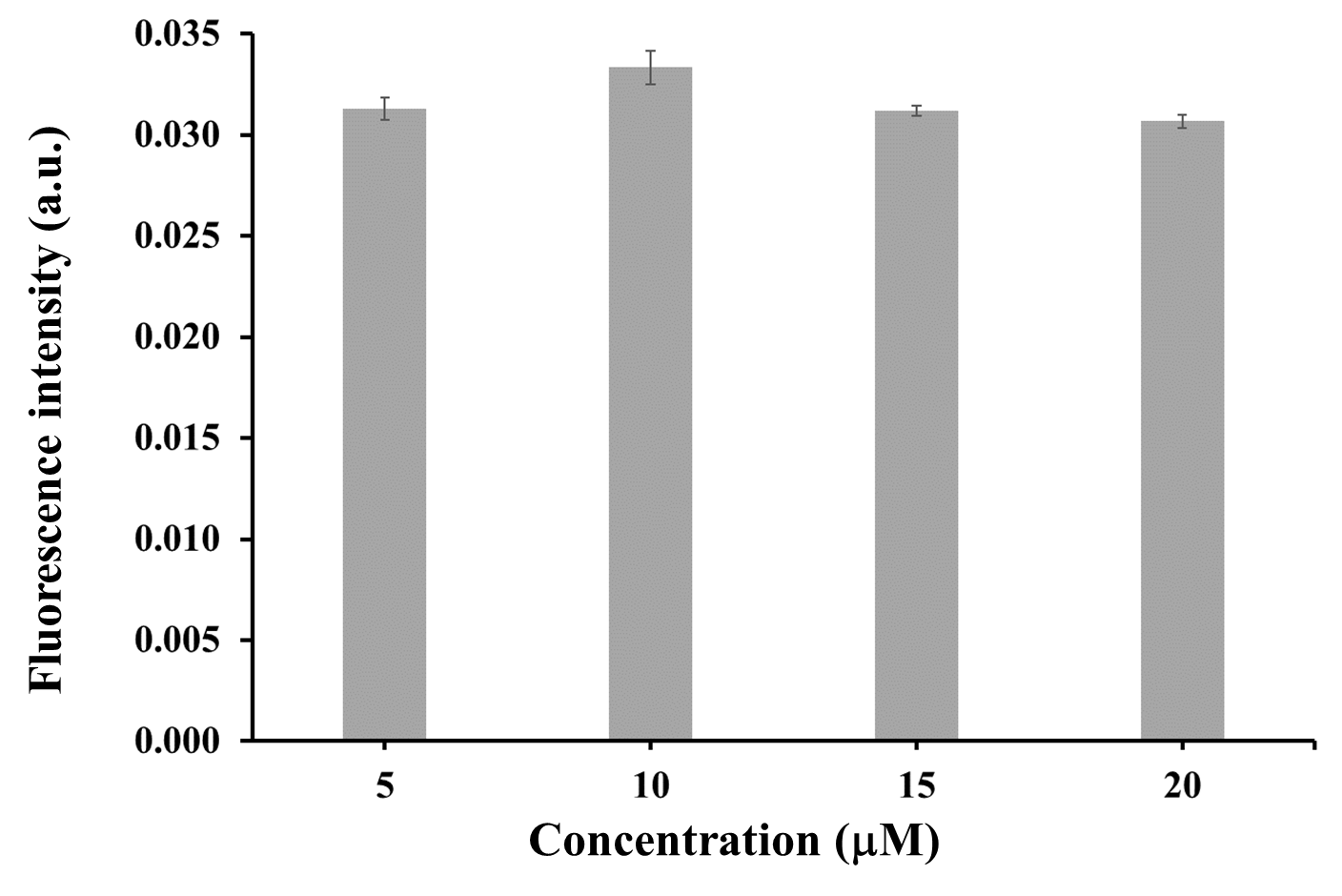

Supplement: S3 Fig — To determine Dox loading, the molar ratio of AS-T9/U4_MH to Dox was fixed at 1:0.095. Measurements were taken at different concentrations (5, 10, 15, and 20 μM) of AS-T9/U4_MH, with corresponding Dox concentrations of 0.475, 0.95, 1.9, and 3.8 μM, respectively. The loading efficiency (LE) was calculated using following equation: LE=Ci−CrCix100 where Ci is an initial Dox concentration, and Cr is the remaining Dox concentration after intercalation. (TIF) [file pone.0317559.s003.tif]

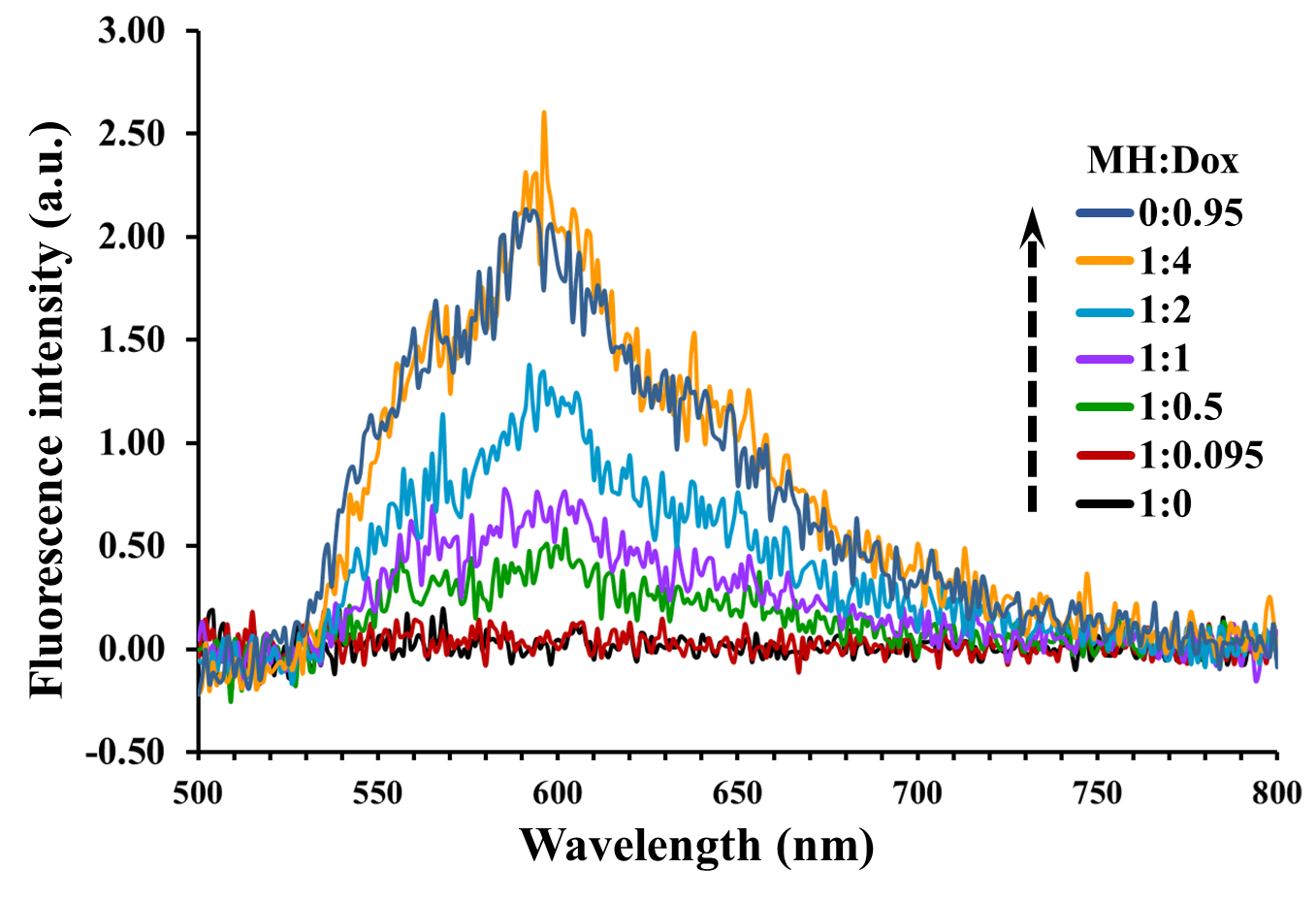

Supplement: S4 Fig — To assess the capacity of AS-T9/U4_MH, 10 μM of AS-T9/U4_MH was incubated with varying concentrations of Dox (0, 0.95, 5, 10, 20, and 40 μM) in PBS solution at room temperature for 1.5 h. The fluorescence intensity of Dox was then measured using a Virokcan microplate reader. (TIF) [file pone.0317559.s004.tif]

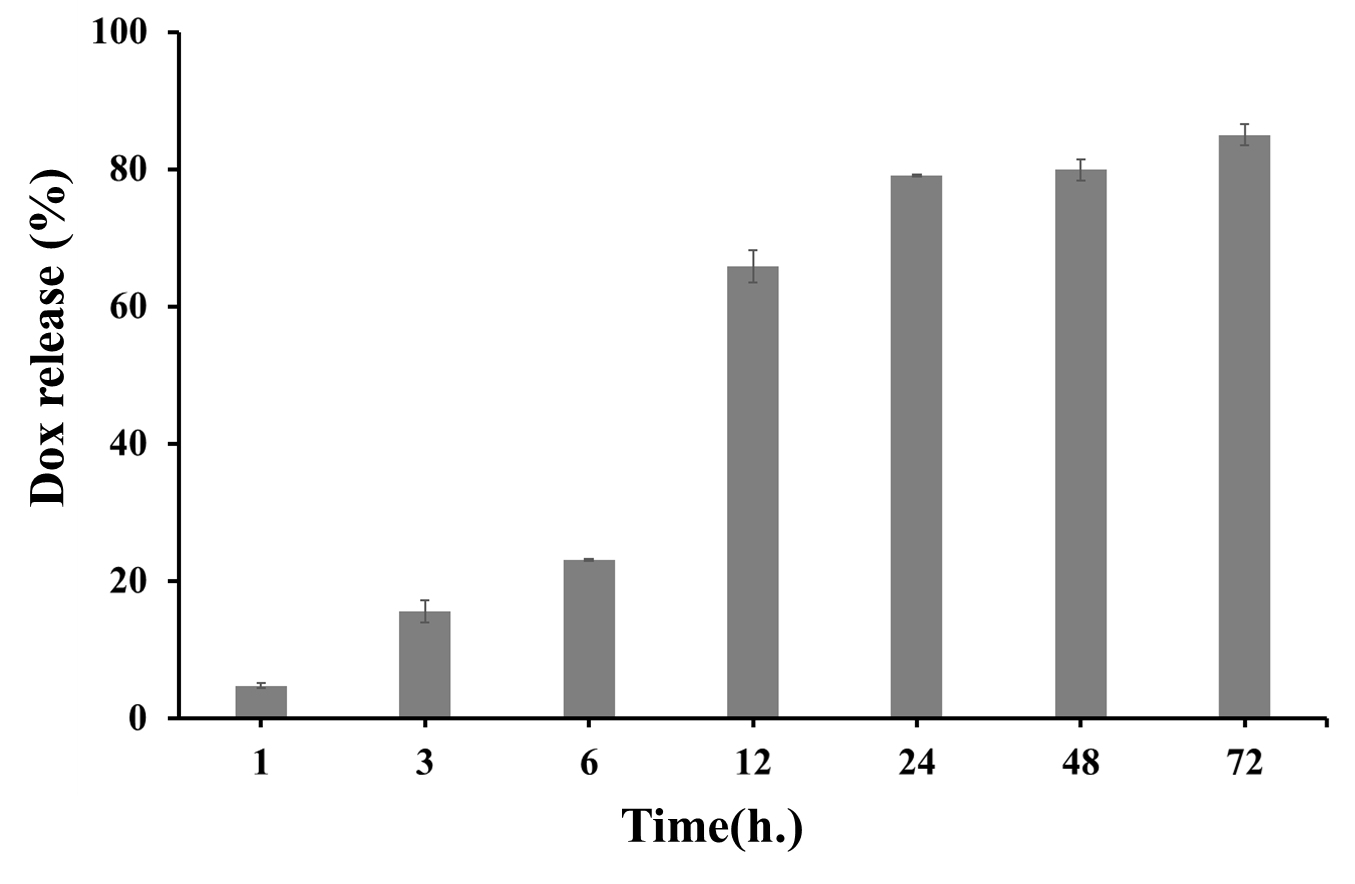

Supplement: S5 Fig — The following experiment measured the amount of Dox released from AS-T9/U4_MH. Dox-loaded AS-T9/U4_MH was incubated in cell culture media at 37°C. Samples were collected at 1, 3, 6, 12, 24, 48, and 72 hours, and the absorption was scanned over a wavelength range of 350 to 800 nm. The maximum absorption peak at 409 nm was used to calculate the percentage of Dox release. A concentration of 0.95 μM of Dox is considered 100%. (TIF) [file pone.0317559.s005.tif]

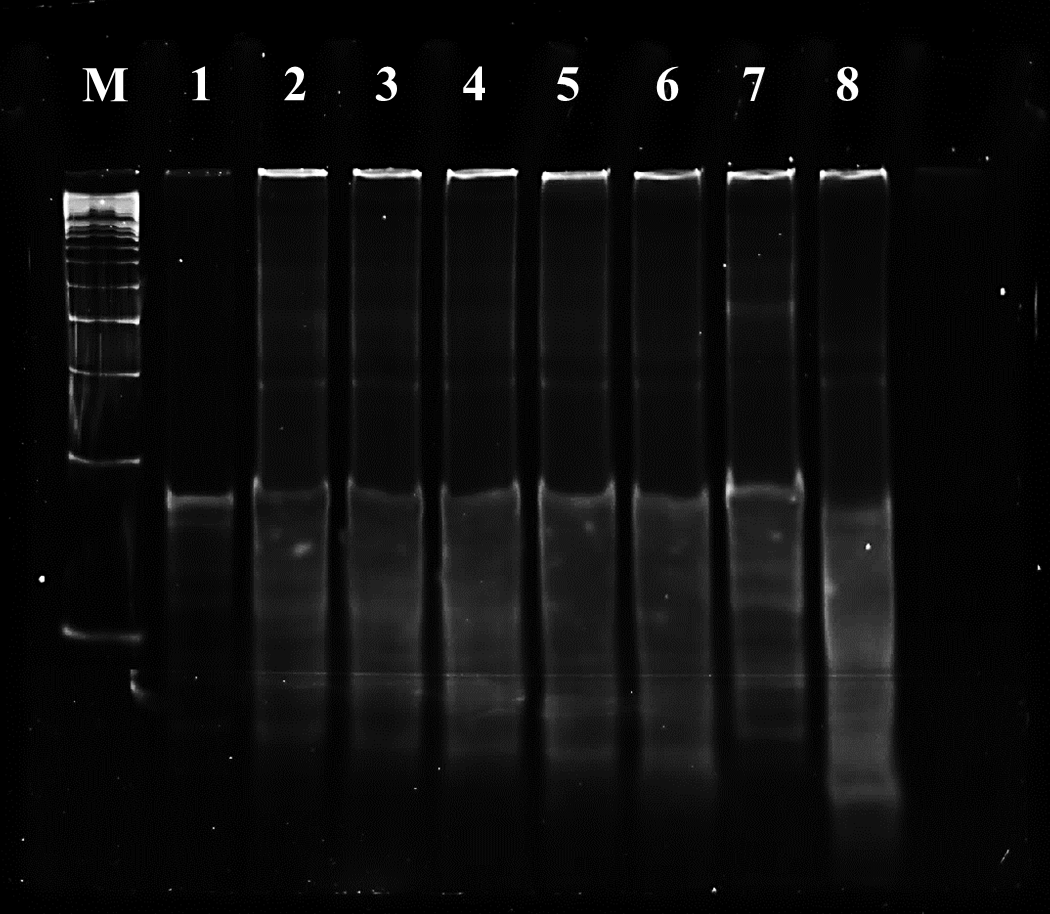

Supplement: S6 Fig — Electrophoresis results of Dox-loaded AS-T9/U4_MH after incubation in cell culture media, with samples collected at various time points. Lane M represents the DNA marker, lane 1 is the Dox-loaded AS-T9/U4_MH, and lanes 2 through 8 correspond to sampling times at 1 h, 3 h, 6 h, 12 h, 24 h, 48 h, and 72 h, respectively. (TIF) [file pone.0317559.s006.tif]

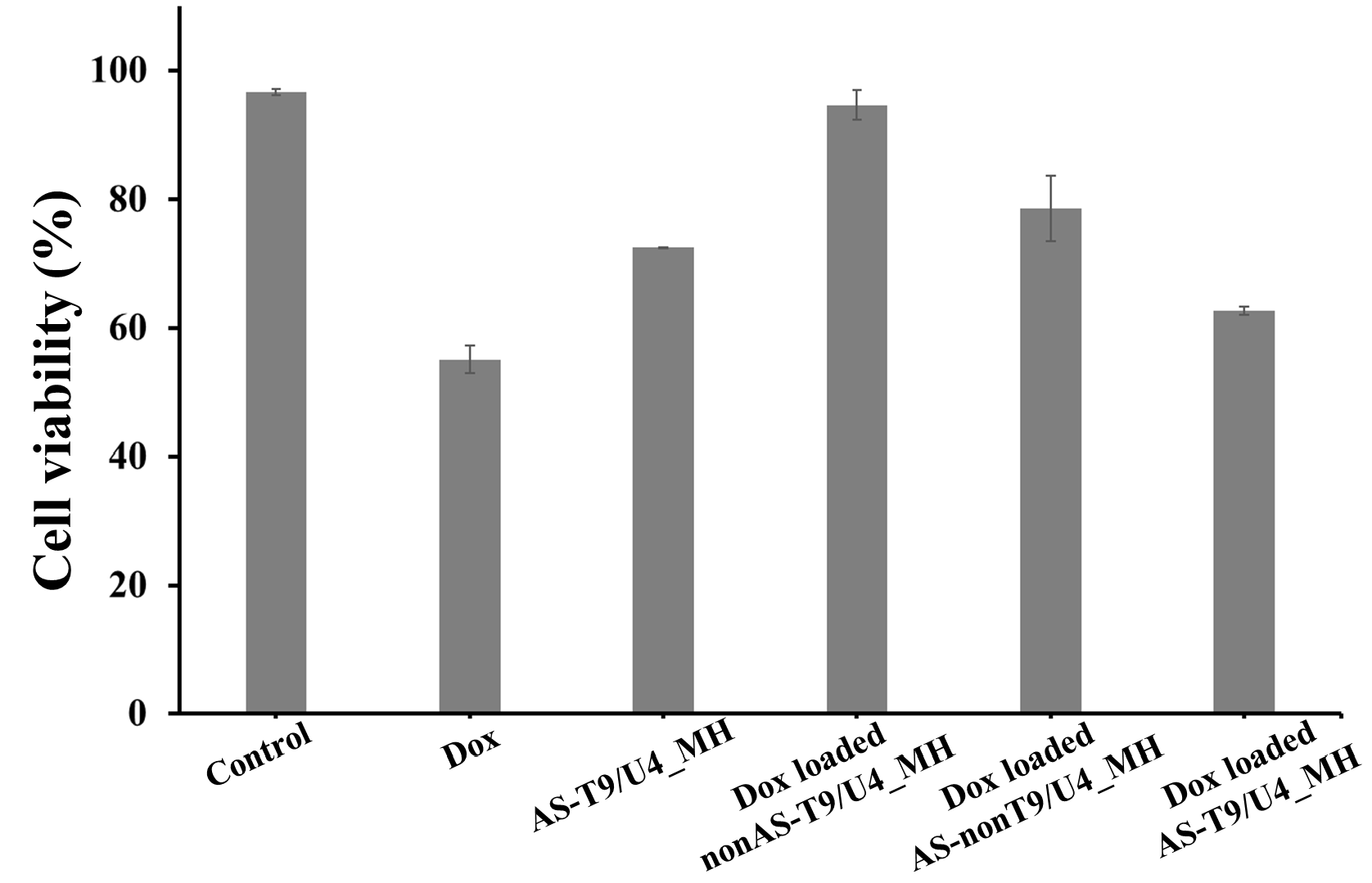

Supplement: S7 Fig — To evaluated the effect of Dox and T9/U4 ASO on SW480 cells proliferation, cells were seeded in 12-well plate at density 2x10^6^ and incubated 24h. After that the cells were treated with 10 μM of AS-T9/U4_MH, Dox-loaded nonAS-T9/U4_MH, Dox-loaded AS-nonT9/U4_MH and Dox-loaded AS-T9/U4_MH for 48 h. After treatment, the cells were collected and staned with trypan blue. Subsequently, the cells were imaged using a microscope (Nikon eclipse ts2r). (TIF) [file pone.0317559.s007.tif]
