# Supplementary material for: Multifunctional molecular hybrid for targeted colorectal cancer cells: Integrating doxorubicin, AS1411 aptamer, and T9/U4 ASO
Source: PLoS One. 2025 Feb 13;20(2):e0317559. doi: 10.1371/journal.pone.0317559 (PMC11825018; doi:10.1371/journal.pone.0317559)

Blot resutlts were imaged using Odyssey LI-COR. The membranes were exposed at 700 nm for 2 min before visualization. The membranes were exposed at 800 nm for 10 min to capture the images.

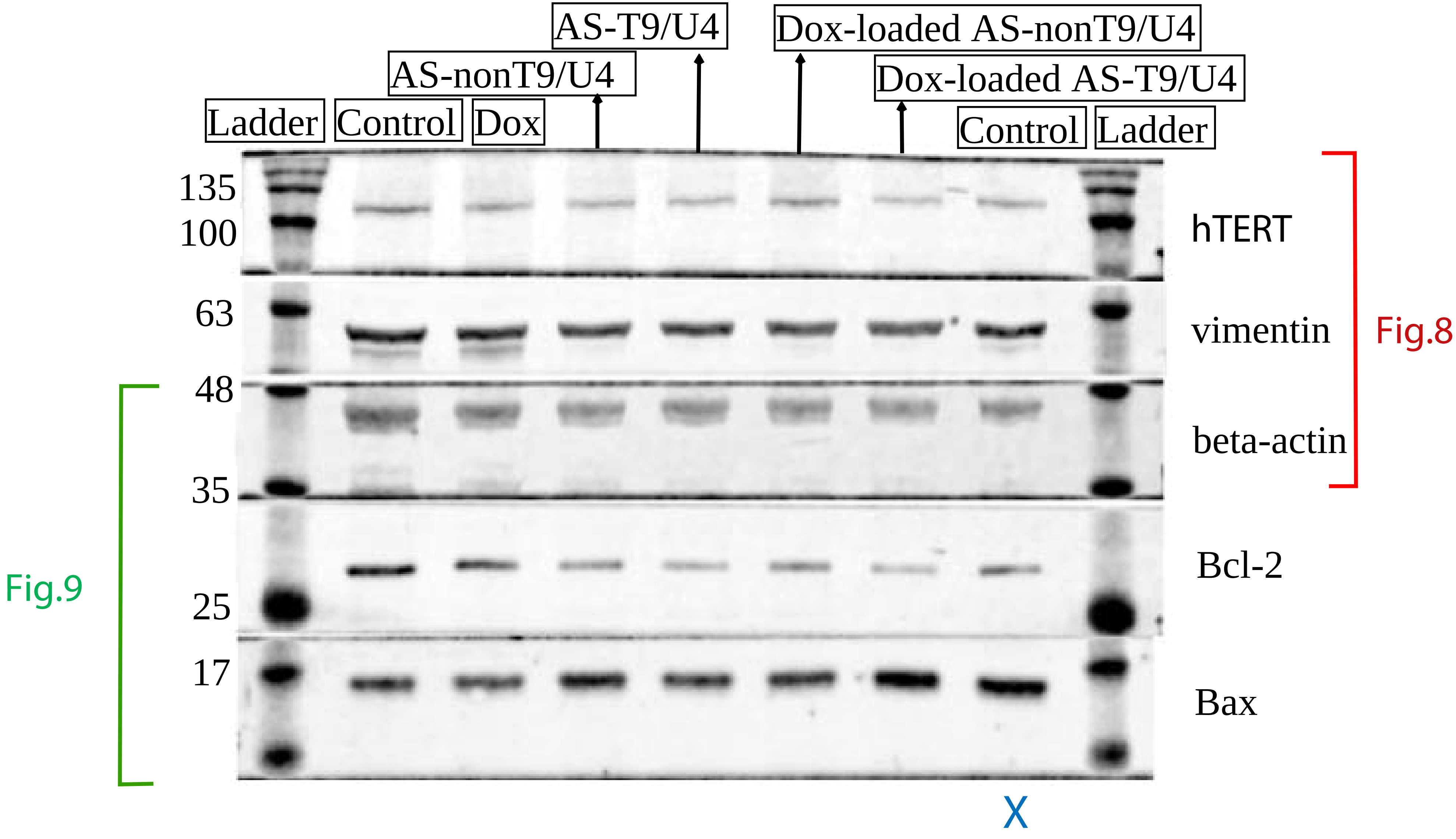

Gel image shown in Fig. 1 was visualized by Gel document (Biorad). The gel was stained with ethidium bromide and exposed to UV light provided by the system.

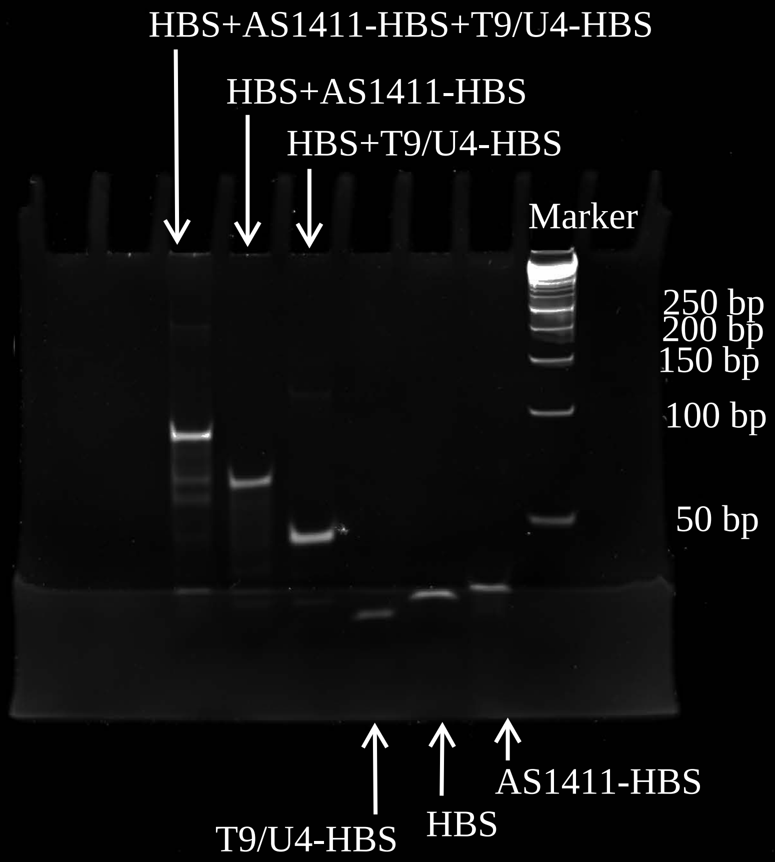

Gel image presented in S6 Fig. was visualized uisg the same GelDoc system as applied for Fig. 1. Dox-loaded AS-T9/U4_MH was incubated in cell culture media at 37^{0}C for 0, 1, 3, 6, 12, 24, 48, and 72 h. before detecting Dox released from the molecular hybrid.

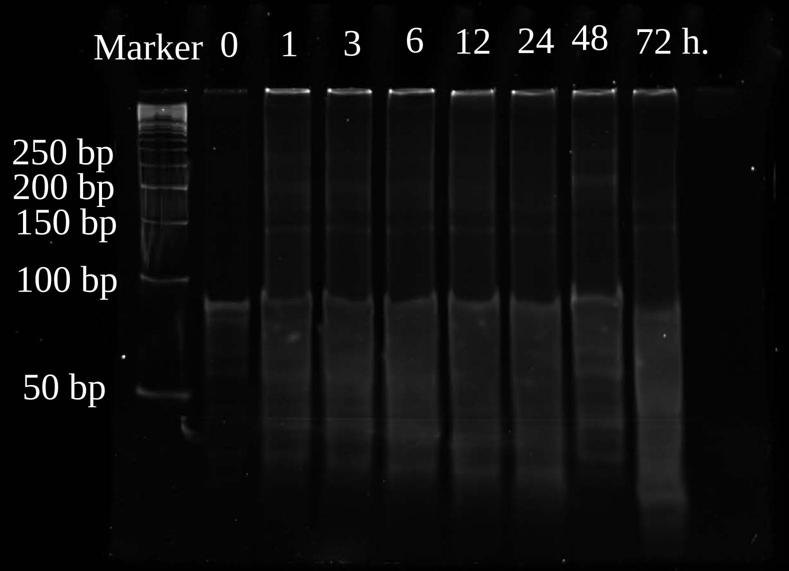

Supplement: S1 Raw images — (PDF) [file pone.0317559.s008.pdf]
