# Supplementary material for: Multifunctional molecular hybrid for targeted colorectal cancer cells: Integrating doxorubicin, AS1411 aptamer, and T9/U4 ASO
Source: PLoS One. 2025 Feb 13;20(2):e0317559. doi: 10.1371/journal.pone.0317559 (PMC11825018; doi:10.1371/journal.pone.0317559)
Supplement: Attachment — Submitted filename: Reviewer response_PLos one-3.docx [file pone.0317559.s009.docx]

**Reviewer #1:**

1. Please note the rigor of the wording, the abstract mentions that “existing treatment approaches encounter challenges related to specificity and efficacy”, while none of the materials selected in this work reflects its specificity to colon cancer, but only the general treatment methods in mediating cancer cell death. Therefore, this study does not solve the inherent problem of 'specificity' in existing treatments. Please revise to avoid ambiguity for the reader.

**Response:** As suggested, we revised the ambiguous statement as follow. “However, current treatment approaches face challenges due to their impact on both cancerous and normal cells, affecting overall efficacy.” (Abstract: Line 18-19)

2. In the validation part of the cell experiment, did the researchers choose other types of cancer cells to prove that the strategy is more therapeutic for colorectal cancer?

**Response:** We appreciate the reviewer’s critical comment on our study. The primary objective of this research was to demonstrate the potential of our MH platform, composed of the AS1411 aptamer, doxorubicin, and T9/U4 ASO, in specifically targeting cancer cells. Colon cancer remains a significant global public health concern. The effects of our MH platform on various colon cell lines (e.g., SW480, Caco-2, and CCD841 CoN) are sufficient to illustrate its specificity and align with previously published studies (1-3). We fully acknowledge the reviewer’s suggestion to expand our investigation to other cancer cell types, which would undoubtedly contribute valuable insights to the scientific community.

*Reference*

1. Lohlamoh W, Soontornworajit B, Rotkrua P. Anti-Proliferative Effect of Doxorubicin-Loaded AS1411 Aptamer on Colorectal Cancer Cell. Asian Pac J Cancer Prev. 2021;22(7):2209-219. Epub 20210701. doi: 10.31557/apjcp.2021.22.7.2209. PubMed PMID: 34319045; PubMed Central PMCID: PMCPMC8607099.

2. Dean P, Kenny B. Cell-surface nucleolin is sequestered into EPEC microcolonies and may play a role during infection. Microbiology (Reading). 2011;157(Pt 6):1761-7. Epub 20110324. doi: 10.1099/mic.0.047506-0. PubMed PMID: 21436219; PubMed Central PMCID: PMCPMC3167913.

3. Dean P, Scott JA, Knox AA, Quitard S, Watkins NJ, Kenny B. The enteropathogenic E. coli effector EspF targets and disrupts the nucleolus by a process regulated by mitochondrial dysfunction. PLoS Pathog. 2010;6(6):e1000961. Epub 20100624. doi: 10.1371/journal.ppat.1000961. PubMed PMID: 20585567; PubMed Central PMCID: PMCPMC2891835.

3. The cell density of each treatment group in Figure 2 was significantly inconsistent under the bright field vision. Under this premise, it was not meaningful to compare the fluorescence signal, and the results were not convincing. It is recommended to improve the experiment to make it more credible.

**Response:** Microscopic experiment has been revised, and the results are shown Fig 2.

4. The conclusion part is recommended to rewrite. Authors should point out the core advantages of this work compared to other delivery or treatment systems, its existing deficiencies, and the follow-up research directions.

**Response:** As suggested the following statements were included in the conclusion (Line 384-390).

Although the study employed in vitro cell models (SW480, Caco-2, and CCD841 CoN) to evaluate the efficacy of the AS-T9/U4 molecular hybrid (MH), these models do not fully capture the complexity of the in vivo tumor microenvironment. Factors such as tumor heterogeneity, immune system interactions, and the presence of stromal components could influence the MH’s performance in a clinical context. Therefore, further validation using animal models is necessary to confirm these findings. To enhance the translational potential of AS-T9/U4_MH, future research should prioritize in vivo studies to assess pharmacokinetics, biodistribution, tumor targeting, and overall therapeutic efficacy, providing critical insights into the clinical feasibility of this molecular hybrid

5. It is not necessary to show each significant differences with “*” between the groups in every bar chart, and the choice should be made around the purpose and focus of the experiment.

**Response:** We have followed the reviewer's suggestion by placing an asterisk "*" to indicate significant differences in Fig 7, 8, and 9, as required.

**Reviewer #2:**

1. The authors should include high resolution and higher magnification of fluorescence microscope images in the Fig. 2 to better illustrate the cellular uptake of FAM-labeled AS-T9/U4_MH. As the authors described, nucleolin is overexpressed on the surface of SW480 cells, the authors are encouraged to discuss how is FAM-labeled AS-T9/U4_MH internalized.

**Response:** We have reworked on fluorescence images using a confocal laser scanning microscope (CLSM, ZEISS CLS 900) to improve the image quality. The cellular uptake of FAM-labeled AS-T9/U4_MH could be described as follows. When AS1411 aptamer binds to nucleolin, it promotes the internalization of FAM-labeled AS-T9/U4_MH into the cytoplasm and nucleus via macropinocytosis, a common form of endocytosis in cancer cells (1). This process is regulated by a nucleolin-dependent mechanism, which disrupts nucleolin-mediated trafficking and efflux. As a result, the MH-containing aptamer becomes trapped within cancer cells, leading to cell death (1, 2). The explanation was included in the manuscript. (Line 210-213)

1. Bates PJ, Reyes-Reyes EM, Malik MT, Murphy EM, O'Toole MG, Trent JO. G-quadruplex oligonucleotide AS1411 as a cancer-targeting agent: Uses and mechanisms. Biochimica et Biophysica Acta (BBA) - General Subjects. 2017;1861(5, Part B):1414-28.
2. Reyes-Reyes EM, Teng Y, Bates PJ. A new paradigm for aptamer therapeutic AS1411 action: uptake by macropinocytosis and its stimulation by a nucleolin-dependent mechanism. Cancer Res. 2010;70(21):8617-29.

2. The authors should directly use Dox-loaded AS-T9/U4_MH to do the fluorescence microscopy or CLSM since Dox itself is fluorescent.

**Response:** We worked on this experiment as suggested. The result showed that Dox was completely intercalated into the molecular hybrid resulting in fluorescence quenching demonstrated in S1 Fig. This result was included in manuscript in Line 266 and 267.

3. In Fig. 3B, the fluorescence intensity in Caco-2 treated with nonAS-T9/U4_MH or AS-T9/U4_MH is higher than that in SW480 treated under the same conditions. The authors should explain this conflicting result.

**Response:** This conflict results from the non-specific binding of Caco-2 on cell surface. So, it shown the high fluorescence intensity more than SW480 in treated cell. But when we compare the only cells treated with FAM-labeled AS-T9/U4_MH and FAM-labeled nonAS-T9/U4 in Caco-2 there are no different peak shifts between them.

4. What is the loading efficiency of Dox?

**Response:** We have carried out a standard curve of Dox fluorescence to determine Dox loading as follows. Standard curve was created to evaluate the concentration of Dox remaining in the solution after intercalation. Dox solutions at various concentrations, including 0.95, 0.48, 0.24, 0.12, 0.06, 0.03, and 0.015 µM, were prepared. The fluorescence intensity at 590 nm was measured when excited at 480 nm using a Virokcan microplate reader. The fluorescence intensity of Dox was measured to evaluate drug loading into AS-T9/U4_MH using a calibration curve method (S2 and S3 Fig.). The findings indicated that AS-T9/U4_MH could encapsulate approximately 98% of Dox within the complex. Additionally, the Dox loading capacity of AS-T9/U4_MH was assessed, as shown in S4 Fig, revealing that as the concentration of Dox increased, the fluorescence intensity also rose. When the molar ratio of AS-T9/U4_MH to Dox was 1:1, the fluorescence intensity of Dox nearly reached its minimum, suggesting that the loading capacity of our MH was a 1:1 molar ratio. (Line 269 – 274 in main manuscript, and Line 637-647 in Supplementary Information)

5. 1% TWEEN 20 in TBS is extremely harsh in doing WB. Some proteins may get washed during the membrane washing step.

**Response:** The concentration of Tween 20 contained a typo, so we have corrected it to 0.1%. (Line 178 and 180)

**Reviewer #3:**

1 It is better to transfer the Cell Culture section (Lines 156-160) after the Evaluation of Stability and Binding of MH in vitro section (Line 131)

**Response:** We rearranged the sections as suggested by the reviewer.

2 The duration of treatment with different compounds should be given in the Effect of Dox-loaded AS-T9/U4_MH on Cell Proliferation section (Line 166).

**Response:** We included the duration of treatment in Line 161.

3 Considering that only two molecular hybrids (FAM-labeled nonAS-T9/U4_MH and FAM-labeled AS-T9/U4_MH) have been examined in the flow cytometry method to check the intensity of fluorescence in the treated cells, other should be replaced with another molecular hybrid in the sentence (Lines 233-234) "AS-T9/U4_MH treatment notably resulted in a significantly detected fluorescent intensity in SW480 cells compared to other molecular hybrids (Figs 3A and 3B).

**Response:** Your comment on writing clarity is acknowledged. We edited our statements as follows. “Flow cytometry analysis was employed to evaluate the intracellular fluorescent intensity subsequent to the treatment of SW480, Caco-2, and CCD841 CoN cells with FAM-labeled nonAS-T9/U4_MH and FAM-labeled AS-T9/U4_MH. FAM-labeled AS-T9/U4_MH treatment notably resulted in a significantly detected fluorescent intensity in SW480 cells compared to FAM-labeled nonAS-T9/U4_MH (Figs 3A and 3B).” (Line 229-232)

3 Error bars should be shown in Figure 3B. Why the increased fluorescence intensity of CaCo-2 cells-treated with molecular hybrids (FAM-labeled nonAS-T9/U4_MH and FAM-labeled AS-T9/U4_MH) compared to the control cells is greater than those of SW480 cells?

**Response:** We added the error bar in Fig 3B. Although the fluorescence intensity CaCo-2 cells treated with molecular hybrids is higher than that of SW480 cells compared to the control, the flow cytometry histogram (Fig 3A) shows minimal differences in fluorescence between cells treated with the MH formulation containing either the AS1411 aptamer or the non-binding sequence. This is likely due to non-specific binding between the tested cells and the applied formulation, a common occurrence in biological assays. This explanation was included in the manuscript. (Line 232-235)

*Reference*

Kalimuthu K, Lubin BC, Bazylevich A, Gellerman G, Shpilberg O, Luboshits G, et al. Gold nanoparticles stabilize peptide-drug-conjugates for sustained targeted drug delivery to cancer cells. Journal of Nanobiotechnology. 2018;16(1). doi: 10.1186/s12951-018-0362-1.

4. As evident in Fig. 3, treatment with the nonAS-T9/U4_MH resulted in an increase in the fluorescence intensity for all three cell types. This observation indicates the cellular uptake of this molecular hybrid containing T9/U4 antisense. Why it had no inhibitory effect on the cell viability (Fig 4)? Also, it is expected that the DOX-loaded nonAS-T9/U4-MH has cytotoxicity against CaCo-2 cells due to the presence of both DOX and T9/U4 antisense. Please interpret it.

**Response:** We are grateful for the reviewer's insightful comment, which has enabled us to further interpret findings.

The results shown in Figures 3 and 4 provide key evidence that the AS1411 aptamer is essential for the MH platform, as it facilitates the cellular uptake of MH. When the AS1411 aptamer binds to nucleolin, it enhances the internalization of Dox-loaded AS-T9/U4_MH into the cytoplasm and nucleus through macropinocytosis, a common endocytic pathway in cancer cells (1). This process is regulated by a nucleolin-dependent mechanism, which disrupts nucleolin-mediated trafficking and efflux. Consequently, the MH complex containing the aptamer, Dox, and ASO becomes trapped inside the cancer cells, leading to cell death (1,2). In the absence of this aptamer or nucleolin, the binding between MHs and the target cells is non-specific, resulting in reduced cellular uptake and diminished anti-proliferative effects.

*References*

1. Bates PJ, Reyes-Reyes EM, Malik MT, Murphy EM, O'Toole MG, Trent JO. G-quadruplex oligonucleotide AS1411 as a cancer-targeting agent: Uses and mechanisms. Biochimica et Biophysica Acta (BBA) - General Subjects. 2017;1861(5, Part B):1414-28.
2. Reyes-Reyes EM, Teng Y, Bates PJ. A new paradigm for aptamer therapeutic AS1411 action: uptake by macropinocytosis and its stimulation by a nucleolin-dependent mechanism. Cancer Res. 2010;70(21):8617-29.

5 For intercalating experiment, the concentration of free DOX and MHs were 0.95 µM and 10 µM, respectively. Please explain why did you choose 0.95 µM for DOX. According to Figure 5, DOX is intercalated in all molecular hybrids and there is no difference between them; therefore, all of molecular hybrids can be used for DOX delivery.

**Response:** The Dox concentration was applied based on its IC_50_ value reported in a previous study, as mentioned in the manuscript (Line 151). The IC_50_ value of Dox represents the concentration at which cell viability is reduced by 50%, providing a benchmark to assess whether the molecular hybrids (MHs) enhance treatment efficacy by further reducing cell viability or mitigate toxicity by increasing it. Since Dox was fully intercalated within the MH formulation, it demonstrated the capability of the MH platform to integrate both recognition elements (e.g., aptamers) and therapeutic elements (e.g., Dox and ASO) into a single molecular hybrid.

*Reference*

Lohlamoh W, Soontornworajit B, Rotkrua P. Anti-Proliferative Effect of Doxorubicin-Loaded AS1411 Aptamer on Colorectal Cancer Cell. Asian Pac J Cancer Prev. 2021;22(7):2209-219. Epub 20210701. doi: 10.31557/apjcp.2021.22.7.2209. PubMed PMID: 34319045; PubMed Central PMCID: PMCPMC8607099.

6 What was the amount of DOX loaded on each of the molecular hybrids? It is better to measure the cumulative amount of DOX release from molecular hybrids over 48 h for interpreting the results of Fig. 6.

**Response:** This comment could help us to improve our study. We further carried out an experiment to quantify Dox loading efficiency, and a capacity of MH (S2, S3 and S4 Figs). The following details and results (S5 Fig) are included in the supplementary information and manuscript (Line 269-277).

Supplementary information

**S2 Fig. A standard curve of Dox.** Standard curve was created to evaluate the concentration of Dox remaining in the solution after intercalation. Dox solutions at various concentrations, including 0.95, 0.48, 0.24, 0.12, 0.06, 0.03, and 0.015 µM, were prepared. The fluorescence intensity at 590 nm was measured when excited at 480 nm using a Virokcan microplate reader.

**S3 Fig. Dox loading.** To determine Dox loading, the molar ratio of AS-T9/U4_MH to Dox was fixed at 1:0.095. Measurements were taken at different concentrations (5, 10, 15, and 20 µM) of AS-T9/U4_MH, with corresponding Dox concentrations of 0.475, 0.95, 1.9, and 3.8 µM, respectively.

**S4 Fig. AS-T9/U4_MH capacity for Dox loading.** To assess the capacity of AS-T9/U4_MH, 10 µM of AS-T9/U4_MH was incubated with varying concentrations of Dox (0, 0.95, 5, 10, 20, and 40 µM) in PBS solution at room temperature for 1.5 h. The fluorescence intensity of Dox was then measured using a Virokcan microplate reader.

**S5 Fig.** **Dox released.** The following experiment measured the amount of Dox released from AS-T9/U4_MH. Dox-loaded AS-T9/U4_MH was incubated in cell culture media at 37°C. Samples were collected at 1, 3, 6, 12, 24, 48, and 72 hours, and the absorption was scanned over a wavelength range of 350 to 800 nm. The maximum absorption peak at 409 nm was used to calculate the percentage of Dox release. A concentration of 0.95 µM of Dox is considered 100%.

(Line 269-274)

The fluorescence intensity of Dox was measured to evaluate drug loading into AS-T9/U4_MH using a calibration curve method (S2 and S3 Fig). The findings indicated that AS-T9/U4_MH could encapsulate approximately 98% of Dox within the complex. Additionally, the Dox loading capacity of AS-T9/U4_MH was assessed, as shown in S4 Fig, revealing that as the concentration of Dox increased, the fluorescence intensity also rose. When the molar ratio of AS-T9/U4_MH to Dox was 1:1, the fluorescence intensity of Dox nearly reached its minimum, suggesting that the loading capacity of our MH was a 1:1 molar ratio.

**Reviewer #4:**

1. To establish MH's advantages over Dox alone, the authors should demonstrate targeted delivery, possibly by using immunofluorescence staining to show colocalization with nucleolin.

**Response:** We appreciate the reviewer's valuable suggestion, which prompted further investigation. However, our results demonstrate that the AS1411 aptamer is critical to the MH platform, as it facilitates the cellular uptake of MH. Upon binding to nucleolin, the AS1411 aptamer enhances the internalization of Dox-loaded AS-T9/U4_MH into the cytoplasm and nucleus via macropinocytosis, a common endocytic pathway in cancer cells. This nucleolin-dependent mechanism disrupts nucleolin-mediated trafficking and efflux, causing the MH complex, which includes the aptamer, Dox, and ASO, to become trapped inside cancer cells, ultimately leading to cell death. Without the aptamer or nucleolin, the binding of MHs to target cells becomes non-specific, reducing cellular uptake and weakening the anti-proliferative effects. As noted in the literature, nucleolin is abundant in SW480 cells but less detectable in Caco-2 and CCD841 CoN cells, supporting our findings and interpretations.

*References:*

Lohlamoh W, Soontornworajit B, Rotkrua P. Anti-Proliferative Effect of Doxorubicin-Loaded AS1411 Aptamer on Colorectal Cancer Cell. Asian Pac J Cancer Prev. 2021;22(7):2209-219. Epub 20210701. doi: 10.31557/apjcp.2021.22.7.2209. PubMed PMID: 34319045; PubMed Central PMCID: PMCPMC8607099.

Dean P, Kenny B. Cell-surface nucleolin is sequestered into EPEC microcolonies and may play a role during infection. Microbiology (Reading). 2011;157(Pt 6):1761-7. Epub 20110324. doi: 10.1099/mic.0.047506-0. PubMed PMID: 21436219; PubMed Central PMCID: PMCPMC3167913.

Dean P, Scott JA, Knox AA, Quitard S, Watkins NJ, Kenny B. The enteropathogenic E. coli effector EspF targets and disrupts the nucleolus by a process regulated by mitochondrial dysfunction. PLoS Pathog. 2010;6(6):e1000961. Epub 20100624. doi: 10.1371/journal.ppat.1000961. PubMed PMID: 20585567; PubMed Central PMCID: PMCPMC2891835.

2. While Dox-loaded AS-T9/U4_MH shows synergetic anti-proliferative effects on colorectal cancer cells, its efficacy is less than free Dox. The authors need to discuss whether this reduced toxicity affects its potential as a cancer treatment

**Response:** We appreciate the reviewer’s insightful comment, which has helped us enhance our findings. The discussion has been incorporated into the manuscript accordingly. (Line 300-304)

While our MH system showed reduced toxicity, its effective concentration was 10 µM, significantly lower than the reported IC_50_ value of 144 µM for the AS1411 aptamer (1). In comparison to other anticancer formulations based on aptamer-DNA nano-assemblies, which typically range between 3-30 µM (2, 3), these findings suggest that our MH formulation holds promise as a potential cancer treatment.

*References*

1.	Lohlamoh W, Soontornworajit B, Rotkrua P. Anti-Proliferative Effect of Doxorubicin-Loaded AS1411 Aptamer on Colorectal Cancer Cell. Asian Pacific Journal of Cancer Prevention. 2021;22(7):2209-19. doi: 10.31557/APJCP.2021.22.7.2209.

2.	Thorat ND, Bauer J, Tofail SAM, Gascón Pérez V, Bohara RA, Yadav HM. Silica nano supra-assembly for the targeted delivery of therapeutic cargo to overcome chemoresistance in cancer. Colloids and Surfaces B: Biointerfaces. 2020;185. doi: 10.1016/j.colsurfb.2019.110571.

3.	Zhang B, Jiang J, Wu P, Zou J, Le J, Lin J, et al. A smart dual-drug nanosystem based on co-assembly of plant and food-derived natural products for synergistic HCC immunotherapy. Acta Pharmaceutica Sinica B. 2021;11(1):246-57. doi: 10.1016/j.apsb.2020.07.026.

3. The manuscript lacks pharmacokinetic characterization. The authors should investigate how the cellular environment affects MH assembly.

**Response:** To investigate how the cellular environment affects MH assembly, two following assays were carried out.

*1) Dox released*. The experiment measured the amount of Dox released from AS-T9/U4_MH. Dox-loaded AS-T9/U4_MH was incubated in cell culture media previously used for SW480 cell culture at 37°C. Samples were collected at 1, 3, 6, 12, 24, 48, and 72 hours, and the absorption was scanned over a wavelength range of 350 to 800 nm. The maximum absorption peak at 409 nm was used to calculate the percentage of Dox release. A concentration of 0.95 µM of Dox is considered 100%. The result was calculated at the maximum absorption peak of 409 nm, which corresponded to a protonated tautomeric form of Dox in acidic solution. At 48 and 72 h, Dox release from the complex reached up to 80% and 85%, respectively (S5 Fig). (Line 274-277)

2) The stability of MH in the cell culture environment was assessed. At specified time points, the released solutions were analyzed using gel electrophoresis. The gel image revealed a gradual decrease in the intensity of the band corresponding to MH as incubation time increased (S6 Fig), suggesting potential disintegration of the MH structure.

**Fig S6** Electrophoresis results of Dox-loaded AS-T9/U4_MH after incubation in cell culture media, with samples collected at various time points. Lane M represents the DNA marker, lane 1 is the Dox-loaded AS-T9/U4_MH, and lanes 2 through 8 correspond to sampling times at 1 h, 3 h, 6 h, 12 h, 24 h, 48 h, and 72 h, respectively.

4. The authors should discuss the study's limitations and future directions.

**Response:** As recommended, the study's limitations and future directions have been incorporated into the manuscript as follows. (Line 384-390)

Although the study employed in vitro cell models (SW480, Caco-2, and CCD841 CoN) to evaluate the efficacy of the AS-T9/U4 molecular hybrid (MH), these models do not fully capture the complexity of the in vivo tumor microenvironment. Factors such as tumor heterogeneity, immune system interactions, and the presence of stromal components could influence the MH’s performance in a clinical context. Therefore, further validation using animal models is necessary to confirm these findings. To enhance the translational potential of AS-T9/U4_MH, future research should prioritize in vivo studies to assess pharmacokinetics, biodistribution, tumor targeting, and overall therapeutic efficacy, providing critical insights into the clinical feasibility of this molecular hybrid.
