# Supplementary material for: Multifunctional molecular hybrid for targeted colorectal cancer cells: Integrating doxorubicin, AS1411 aptamer, and T9/U4 ASO
Source: PLoS One. 2025 Feb 13;20(2):e0317559. doi: 10.1371/journal.pone.0317559 (PMC11825018; doi:10.1371/journal.pone.0317559)
Supplement: Attachment — Submitted filename: Response to reviewer_Molecular hybrid-1.docx [file pone.0317559.s010.docx]

Reviewer #1: This work studied the potential anticancer acitivity of a multifunctional molecular hybrid which integrates doxorubicin, AS1411 aptamer, and T9/U4 ASO. The general research idea and the experimental strategy are reasonable. However, the editing quality of manuscript were bad. For example, the sections of abstract and introduction are too verbose. Many well-known contents do not require detailed elaboration.

Response: As suggested, abstract and introduction are revised. (Line 22-24, 34-35, 38-40, 44-48, 52-53, 84-86)

Reviewer #2:

The manuscript by Boonchoy Soontornworajit et. al reported the design of a molecular hybrid (MH) containing doxorubicin, AS1411 aptamer, and T9/U4 ASO for the selective inhibition of nucleolin overexpressed colorectal cancer cells. The authors addressed most of the questions from the previous reviewers, but the significance of the design is still not well established because of the low anticancer efficacy. Therefore, I do not recommend accepting this paper until the advantages of the design are clearly demonstrated and some major issue get addressed.

**Response:** The effect of AS-T9/U4_MH and Dox-loaded AS-T9/U4_MH on cell proliferation was evaluated using the MTS assay. However, the comparison between MH with and without Dox was not included in the previous manuscript submission. To address this, we analyzed and compared the cell viability levels in cells treated with AS-T9/U4_MH and Dox-loaded AS-T9/U4_MH, and the findings have been incorporated into the revised manuscript (Fig. 6). The results demonstrated a significant difference in cell viability between these two groups, highlighting the enhanced antiproliferative effect resulting from Dox incorporation in the MH (Line 146, 278-280, 300). To further emphasize the efficacy of the MH in suppressing cell proliferation, a trypan blue assay was performed and included in the revised manuscript (S7 Fig) (Line 601-605). The results revealed that SW480 cells treated with Dox-loaded AS-T9/U4 exhibited significantly greater antiproliferative effects compared to AS-T9/U4, Dox-loaded nonAS-T9/U4, and Dox-loaded AS-nonT9/U4. These findings are consistent with the MTS assay results. The trypan blue assay provided a clear distinction between live and dead cells based on membrane integrity, further supporting the effectiveness of the MH (Line 282-286).

1. The author stated that when molar ratio of AS-T9/U4 MH to Dox reaches 1:1, the fluorescence intensity reaches minimum, which is inconsistent with S4 Fig. In S4 Fig, 1:0.095 is the minimum, which is 10:1. And the calculation of loading efficiency is still unclear.

**Response:** From the manuscript, “Additionally, the Dox loading capacity of AS-T9/U4_MH was assessed, as shown in S4 Fig, revealing that as the concentration of Dox increased, the fluorescence intensity also rose. When the molar ratio of AS-T9/U4_MH to Dox was 1:1, the fluorescence intensity of Dox nearly reached its minimum, suggesting that the loading capacity of our MH was a 1:1 molar ratio.” These statements could confuse readers, as they describe how we estimated the Dox loading capacity based on the minimum detectable fluorescence signal. Following the reviewer's comments, we have clarified the ambiguous wording. “When the molar ratio of AS-T9/U4_MH to Dox was 1:1, the fluorescence intensity of Dox nearly reached the minimum detectable signal, suggesting that the loading capacity of our MH was a 1:1 molar ratio.” (Line 256)

In addition, we have carried out a standard curve of Dox fluorescence to determine Dox loading as follows. Standard curve was created to evaluate the concentration of Dox remaining in the solution after intercalation. Dox solutions at various concentrations, including 0.95, 0.48, 0.24, 0.12, 0.06, 0.03, and 0.015 µM, were prepared. The fluorescence intensity at 590 nm was measured when excited at 480 nm using a Virokcan microplate reader. The fluorescence intensity of Dox was measured to evaluate drug loading into AS-T9/U4_MH using a calibration curve method (S2 and S3 Fig.). The loading efficiency (*LE*) was calculated using following equation: $LE=\frac{C_{i}-C_{r}}{C_{i}}x 100$ where *C_i_* is an initial Dox concentration, and *C_r_* is the remaining Dox concentration after intercalation. This information is included in the supporting information (Line 585-587)

2. What is the ratio of T9/U4-HBS, AS1411-HBS, and a hybridization strand when making the molecular hybrid and how to determine the concentration? How to collect molecular hybrid after you making it? How to collect Dox loaded molecular hybrid?

**Response:** To prepare the molecular hybrid (MH), all sequences were used at a concentration of 10 µM, maintaining a 1:1:1 molar ratio of T9/U4-HBS, AS1411-HBS, and the hybridization strand. The gel image (Fig. 1) confirms a single band corresponding to the complete oligonucleotide formulation, indicating that the MH concentration was indeed 10 µM. For Dox-loaded MH preparations, Dox was incorporated at specified concentrations into the 10 µM MH, and these formulations were subsequently analyzed in our study.

3.1 Why did the author use 5 μM of MH in the flow cytometry part instead of using 10 μM as same as other experiment? 3.2 Why did the authors use 10 μM, which is not effective according to the cell viability. The cell viability of SW480 being treated with AS-T9/U4_MH is greater than 70 %. The cell viability of SW480 being treated with Dox loaded AS-T9/U4_MH is greater than 60 %.

**Response:** In the early stages of our study, we used 10 µM of MH in flow cytometry, which revealed non-specific binding in SW480 cells, as shown in **Figure R1**. To address this issue, the MH concentration was reduced to 5 µM for flow cytometry, which effectively minimized non-specific binding while still demonstrating the binding capability of the MH, as illustrated in Fig. 3 of the manuscript. For cell viability assays, we followed our previous publication [1] and used an MH concentration of 10 µM. The cell viability of SW480 cells treated with AS-T9/U4_MH and Dox-loaded AS-T9/U4_MH was approximately 70% and 60%, respectively, with a significant difference that highlights the cytotoxic effect of Dox in the MH (**Figure R2**). Furthermore, these viability levels exceeded the 50% benchmark for Dox at its IC_50_ concentration, demonstrating the MH's advantage in reducing Dox toxicity.

**Figure R1.** Flow cytometry histogram of SW480 and Caco-2 cells treated with 10 μM of FAM-labeled nonAS-T9/U4_MH and FAM-labeled AS-T9/U4_MH at 37 ºC for 1.5 h, and no treatment as a control.

**Figure R2.** The cell viability of SW480 after treated with 10 μM of AS-T9/U4_MH and Dox-loaded AS-T9/U4_MH at 37°C for 48h. *P < 0.05. The data are presented as means ± SD, n = 3.

[1.] Rotkrua P, Lohlamoh W, Watcharapo P, Soontornworajit B. A molecular hybrid comprising AS1411 and PDGF-BB aptamer, cholesterol, and doxorubicin for inhibiting proliferation of SW480 cells. Journal of Molecular Recognition. 2021;34(11):e2926. doi: <https://doi.org/10.1002/jmr.2926>.

4. The great fluorescence increases of Caco-2 cells being treated with FAM-labeled nonAS-T9/U4_MH observed using flow cytometry is not consistent with fluorescence images. Please explain.

**Response:** Several studies discuss the challenges and conflicts between results obtained from flow cytometry and fluorescence microscopy. While these two methods are complementary, discrepancies often arise due to their different approaches to cell analysis. Flow cytometry measures fluorescence intensity at a population level, providing quantitative data but lacking morphological context. In contrast, microscopy captures visual details and individual cell morphology but is limited in statistical robustness. For instance, flow cytometry might detect population-wide changes in fluorescence, while microscopy could highlight cell-specific variability [1, 2]. For example, Domínguez-Fandos et al., when comparing flow cytometry and fluorescence microscopy for analyzing human sperm, noted that various factors can influence the results obtained through flow cytometry. These factors include differences in the methods used to define the target population, apply gating strategies, and calculate the frequency of positive events [3]. In our findings, the inconsistency between microscopy images and flow cytometry histograms observed in Caco-2 cells might be due to differences in population sizes: approximately 1,000 cells analyzed via microscopy compared to 30,000 cells via flow cytometry. Additionally, fluorescence bleaching might significantly affect the microscopic assay.

References

1. Brestoff, J. R.; Frater, J. L. Contemporary Challenges in Clinical Flow Cytometry: Small Samples, Big Data, Little Time. *The Journal of Applied Laboratory Medicine* **2022**, *7* (4), 931-944. DOI: 10.1093/jalm/jfab176.

2. W.L. Godfrey, D.M. Hill, J.A. Kilgore, G.M. Buller, J.A. Bradford, D.R. Gray, I. Clements, K. Oakleaf, J.J. Salisbury, M.J. Ignatius, and M.S. Janes. Complementarity of Flow Cytometry and Fluorescence Microscopy. *Microsc Microanal* **2005**, 11, 246-247.

3. Domınguez-Fandos D, Camejo MI, Ballesca JL, Oliva R. Human sperm DNA fragmentation: correlation of TUNEL results as assessed by flow cytometry and optical microscopy. *Cytometry Part A* **2007**, 71A, 1011–1018.

5. Could the author briefly explain the dissociation mechanism of Dox loaded MH inside cells?

**Response:** After cellular internalization, the Dox-loaded MH releases Dox molecules within SW480 cells, possibly driven by diffusion due to the relatively low intracellular Dox concentrations. Alternatively, the release may occur through the gradual degradation of the aptamer by lysosomal endonucleases following uptake. These mechanisms, as described in the literature, may work together to facilitate Dox dissociation. This explanation was included in the manuscript. (Line 275-278)

References

Bagalkot V, Farokhzad OC, Langer R, Jon S (2006) An aptamer-doxorubicin physical conjugate as a novel targeted drug-delivery platform. Angew Chem Int Ed Engl 45(48):8149–8152.
