# Supplementary material for: Multifunctional molecular hybrid for targeted colorectal cancer cells: Integrating doxorubicin, AS1411 aptamer, and T9/U4 ASO
Source: PLoS One. 2025 Feb 13;20(2):e0317559. doi: 10.1371/journal.pone.0317559 (PMC11825018; doi:10.1371/journal.pone.0317559)
Supplement: Attachment — Submitted filename: Reviewer respone E.3.docx [file pone.0317559.s011.docx]

**Reviewer #2**: The authors have adequately addressed most of the comments from previous reviewers. The overall concept and experimental approach are reasoned and sound. Therefore, I recommend this manuscript for acceptance in PLOS ONE after addressing a few minor issues.

1. Regarding the ratio, although the authors explained that at a 1:1 molar ratio of AS-T9/U4_MH to Dox, the fluorescence intensity of Dox nearly reached the minimum detectable signal, it is unclear why they continued to use 0.95 µM Dox and 10 µM MH in the experiments, resulting in a 1:10 ratio. Additionally, in Figure S3, to determine Dox loading, the molar ratio of MH to Dox was fixed at 1:0.095. The rationale for these choices needs further clarification.

**Response:** The MH formulations presented in this study were designed with two main objectives. First, to evaluate the capability of MHs in cellular assays, including microscopy, flow cytometry, and cell viability tests. For these experiments, the Dox concentration was set at its IC_50_ value of 0.95 µM, while the MH concentration was maintained at 10 µM, based on findings from our previous publication [1]. Second, to assess Dox loading, fluorescence spectroscopy was used to investigate the fluorescence quenching of Dox at varying MH ratios, with the results shown in Figure S3.

[1.] Rotkrua P, Lohlamoh W, Watcharapo P, Soontornworajit B. A molecular hybrid comprising AS1411 and PDGF-BB aptamer, cholesterol, and doxorubicin for inhibiting proliferation of SW480 cells. Journal of Molecular Recognition. 2021;34(11):e2926. doi: <https://doi.org/10.1002/jmr.2926>.

2. In Figure S1, it is unclear why Dox is not released from MH into the cytosol. If Dox were released, fluorescence should be observed. The authors should provide an explanation or additional data to clarify this observation.

**Response:** To evaluate the intercalation of Dox into dsDNA, SW480 cells were treated with Dox-loaded AS-T9/U4_MH for 1.5 hours and subsequently visualized using CLSM. As shown in Figure S1, the results confirm the complete intercalation of Dox within MH. The cellular imaging aligns with the Dox release experiment (Figure S5), which demonstrated that only about 5% of Dox was released from MH after 1 hour. This minimal amount of Dox is likely insufficient to be detected inside the cells using fluorescence microscopy. This explanation was included in the manuscripts (line 260-262)

3. In Figure 6, there is an inconsistency between the colors of the columns and the corresponding captions. The authors should ensure that the colors and captions are accurately aligned to avoid confusion.

**Response:** The colors in Figure 6 were adjusted to match its caption.

**Fig 6.** The cell viability of CCD841 CoN, Caco-2, and SW480 cells after treatments at 48 h of 0.95 μM Dox, 10 μM AS-T9/U4_MH, Dox-loaded nonAS-T9/U4_MH, Dox-load AS-nonT9/U4_MH, Dox-load AS-T9/U4_MH, and no treatment as a control. The values are presented as means ±SD, n = 3, *P < 0.05.

**Reviewer #3**: The authors failed to respond to comments previously raised and the quality of this work still fall behind the criteria of PLOS ONE. Thus, I recommended the rejection of this work.

Response: We appreciate the reviewers' comments.
